# Analysis of the Quasi-Static and Dynamic Fracture of the Silica Refractory Using the Mesoscale Discrete Element Modelling

**DOI:** 10.3390/ma14237376

**Published:** 2021-12-01

**Authors:** Aleksandr S. Grigoriev, Andrey V. Zabolotskiy, Evgeny V. Shilko, Andrey I. Dmitriev, Kirill Andreev

**Affiliations:** 1 Institute of Strength Physics and Materials Science of Siberian Branch of the Russian Academy of Sciences (ISPMS SB RAS), 2/4, pr. Akademicheskii, 634055 Tomsk, Russia; dmitr@ispms.ru; 2 Magnezit Group, 34, St. Solnechnaya, 456910 Satka, Russia; azabolotskiy@magnezit.com; 3 The State Key Laboratory of Refractories and Metallurgy, Wuhan University of Science and Technology (WUST), 947 Heping Ave., Wuhan 430081, China; kpandreev@gmail.com; 4 Ceramic Research Centre, Tata Steel, 211 Rooswijkweg, 1951 MD Velsen Noord, The Netherlands

**Keywords:** refractories, dynamic loading, fracture, mesoscale computer simulation, discrete element method (DEM)

## Abstract

Computer modelling is a key tool in the optimisation and development of ceramic refractories utilised as insulation in high-temperature industrial furnaces and reactors. The paper is devoted to the mesoscale computer modelling of silica refractories using the method of homogeneously deformable discrete elements. Approaches to determine the local mechanical properties of the constituents from the global experimental failure parameters and respective crack trajectories are considered. Simulations of the uniaxial compressive and tensile failure in a wide range of quasi-static and dynamic loading rates (10^2^ s^−1^) are performed. The upper limit of the dynamic loading rates corresponds to the most severe loading rates during the scrap loading on the refractory lining. The dependence of the strength, fracture energy, and brittleness at failure on the loading rate is analysed. The model illustrates that an increase in the loading rate is accompanied by a significant change in the mechanical response of the refractory, including a decrease in the brittleness at failure, a more dispersed failure process, and a higher fraction of the large grain failure. The variation of the grain–matrix interface’s strength has a higher impact on the static compressive than on the static tensile properties of the material, while the material’s dynamic tensile properties are more sensitive to the interface strength than the dynamic compressive properties.

## 1. Introduction

Refractories are ceramic materials utilised to construct industrial furnaces and high-temperature reactors [1,2,3,4,5,6,7,8]. They have a composite microstructure, which typically features large filler grains, a matrix of smaller grains, pores, and micro-cracks. There exist refractories of different chemical compositions and morphology of microstructure to match different service conditions. Their mechanical behaviour is characterised by the quasi-brittle failure and sensitivity of strength to hydrostatic stress [2,3]. Due to similar microstructural set-up and mechanical behaviour, they are related to civil engineering materials, rocks, and soils. In service, refractories are exposed to mechanical loads of diverse types and intensity [2,3], featuring compressive and tensile stresses resulting from the interactions between different parts of the refractory structure and under the influence of the process conditions. The majority of loads are characterised by quasi-static rates. In specific instances, e.g., under the impact of the hydraulic hammer repair works or during the loading of scrap [1,9], dynamic loading occurs. The refractories’ ability to sustain different loads is critical for the reliability and productivity of the high-temperature industrial equipment.

For refractories, microstructural aspects of crack formation and propagation have been attracting major research attention [3,10,11,12,13]. One of the key factors influencing fracture behaviour is the interface between microstructural constituents. The results obtained for several types of refractories depict that the brittleness at failure correlates with an increase of the relative crack length along the grain/matrix interface [14]. In model civil engineering concrete, the samples with weaker interfaces had lower bending strength [15] and brittleness at failure [16]. For composites in general, the strength of the interface between the matrix and reinforcing constituents (large grains, fibres) positively correlates with the material’s strength [17,18,19]. For fracture energy and the brittleness at failure, this relationship has a maximum [17] extremely strong interface that leads to brittle failure. Such observations are made for quasi-static failure. A few existing publications on the dynamic failure of refractories only address the macrostructural aspects of failure [9,20]; thus, little is known about its meso-structural mechanisms.

Apart from classical materials’ science analysis, computer modelling is an efficient tool for the mesoscale analysis of fracture [10,11,21,22,23,24,25,26]. The advantage of numerical computer analysis is the ability to freely vary the properties of different constituents and correlate those with macroscopic mechanical properties. The challenge of mesoscale modelling is the accurate representation of the properties of the constituents forming the material [27,28]. Most studies on refractories are based on generic representation of the microstructure [10,21,22,25,26]. The computer modelling of refractories’ mechanical behaviour is realised using two alternative approaches. The first one is a continuum-based approach, such as finite element method (FEM) [10,21,22,29,30], which allows the representation of complex shapes of constituents and their non-linear behaviour. However, this method has a limitation in the modelling of crack growth [27,31,32]. The widely used algorithm of the “smearing” of real discontinuity (crack), including the phase-field models and smeared crack band models, does not always permit the appropriate simulation of crack localisation, especially under the conditions of unstable crack growth. The advanced XFEM implementations [33,34] require rather higher computational costs and still experience difficulties with crack weaving and branching. Contrary to the continuum-based methods, the discrete approach is perfectly suited to model formation and growth of micro- and macro-cracks [27,35,36,37,38]. Here, the material is represented by the ensemble of bonded finite-sized particles. The formation of a discontinuity is modelled as an extremely localised process by means of separating the surfaces of adjacent particles. For refractories, the discrete element method (DEM) has been applied in a limited number of studies [11,25], in which its classical implementation, treating the particles as undeformable (rigid) volumes, has been used. To the authors’ best knowledge, the computer modelling of the failure of refractories under dynamic loads has not yet been performed.

In the current paper, the fracture of silica refractories was analysed using the mesoscale DEM model. The advanced method of homogeneously deformable DEM was used [39,40,41], which assents to the modelling of crack formation and growth both under quasi-static and dynamic loading. The macroscopic parameters of failure and mesoscopic processes of crack formation under uniaxial compression and tension were examined within the broad interval of strain rates from quasi-static values up to the dynamic rates of 10^2^ s^−1^, specific focus points being the accurate representation of the properties of constituents and analysis of the grain–matrix interface’s influence on the failure of the material.

## 2. Materials and Methods

### 2.1. Material and Laboratory Tests

**Figure 1:**
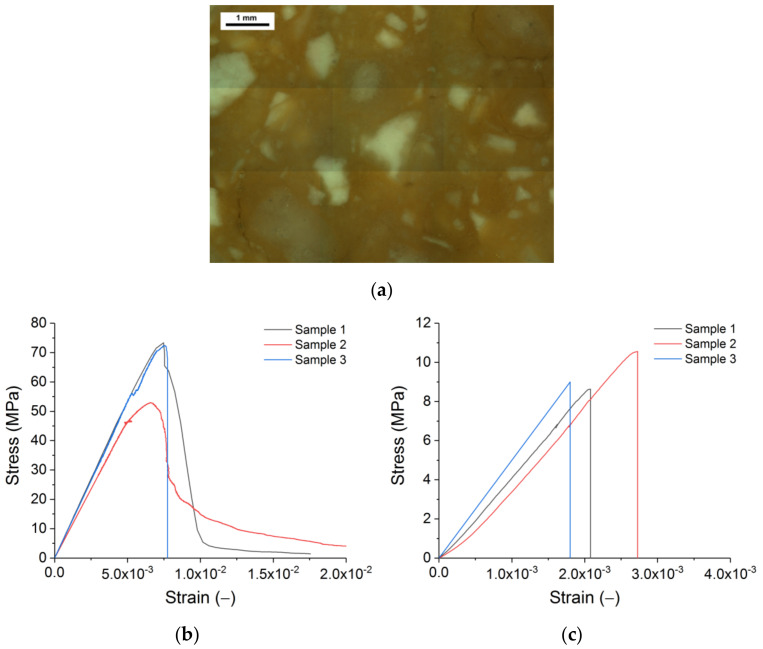
A typical microstructure of silica refractories as seen in light optical microscopy conducted in cross-parallel light (**a**), and experimentally obtained diagrams of a uniaxial compression test (**b**) and a three-point bending test (**c**).

Computer modelling was used to simulate the commercially available silica brick (Figure 1). Such bricks are used to construct coke ovens, heat exchangers of blast furnaces, and glass-making furnaces. Properties of the studied material were broadly discussed in a previous publication [42]. SiO_2_ comprises more than 96 wt % of the material. The main mineralogical phases are tridymite and cristobalite. Tridymite forms the grains of the matrix and the rims of larger grains [12,42]. Cristobalite is predominantly found in large grains. The grain size distribution, as indicated by the supplier, is as follows: 1–2 mm grains are 2 wt %, 0.5–1 mm grains make up 13 wt %, and 0.1–0.5 mm grains comprise 29 wt %; the rest of the grains are finer than 0.088 mm. The bulk and true densities of the brick are 1.84 g/cm^3^ and 2.35 g/cm^3^, respectively. The total porosity is 22%.

Room temperature mechanical properties are described in the following sentences. Dynamic Young´s modulus is 12 GPa [42]. Static Young´s modulus depends on the test set-up. Typically, it can be 2–3 times lower than the dynamic value (Figure 1b,c). The compressive strength is 40–50 MPa (Figure 1b). The typical value of three-point bending strength is 8–10 MPa (Figure 1c). Specific fracture energy under tension is 60 N/m [42]. The number of laboratory samples used to obtain different mechanical properties varied between 3 and 10. For mechanical properties, the typical statistical spread is 10–20% of the average value.

In the paper, images of cracks obtained by us in different laboratory experiments are used to validate the DEM results. In the compressive test, the prismatic samples had a height of 50 mm and a cross-section of 30 × 30 mm^2^. In the three-point bending tests, the geometry was 40 × 40 × 120 mm^3^ with a span of 100 mm. The loading rate was 0.1 mm/min and 0.3 mm/min for the compressive and bending tests, respectively. For the wedge splitting test, the typical geometry [12] and the loading rate of 0.5 mm/min were used. The width, depth, and height of the samples were 100 mm, 65 mm, and 100 mm, respectively. Due to notches, the failed cross-section had the width and height of 55 mm and 66 mm, respectively. The vertical notch was 12 mm. The wedge angle was 8°. In the analysis of the crack propagation through different constituents of the microstructure, 6 crack trajectories of three wedge splitting test samples were analysed. Per sample, two polished sections exposing crack at different depths of the sample were used.

### 2.2. DEM Models

**Figure 2:**
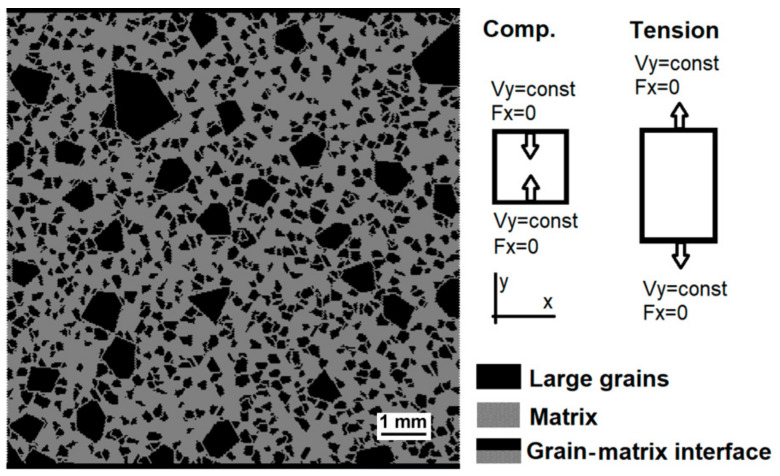
Schematic of DEM models of uniaxial compression and tension. The picture shows the model sample 1 × 1 cm^2^.

Two-dimensional (2D) mesoscopic DEM samples of a representative material volume were constructed (Figure 2), and refractory was considered a particle-reinforced composite. The microstructure is modelled by the discrete element’s ensembles with appropriate mechanical characteristics. The discrete element’s diameter (35 μm) was chosen to ensure that the minimum size of grains exceeded the element diameter by at least three times. A special study for the convergence of the results presented that a decrease in the element diameter does not lead to a significant change in the fracture pattern and integral characteristics of the mechanical response of the samples. The plane stress approximation was used.

The model explicitly takes into consideration the particles of three grain fractions larger than 0.1 mm (1–2 mm, 0.5–1 mm, and 0.1–0.5 mm grains), which were considered non-porous inclusions; their shape qualitatively corresponds to that of the corresponding fractions of a real sample (Figure 1a). The volumetric content of fractions conforms to the volumetric content of similar fractions in a real refractory. The sample regions formed by finer fractions and microscale pores were modelled as homogeneous continual, forming the matrix of the modelled material. We assumed that all the porosity of the refractory is concentrated in the matrix. The spatial placement and determination of the large grains’ geometry in the model were executed out using the Voronoi tessellation algorithm [43]. Representing by the model of the grain size distribution as during the pressing of the bricks ignores the changes of the grain size and shape to possibly occur during the sintering of the bricks.

Uniaxial tension and compression of the samples were modelled. For the compressive and tensile tests, the geometry of the model was 1 × 1 cm^2^ and 1 × 1.5 cm^2^, respectively (Figure 2). For every loading mode, we considered three “statistically equivalent” samples with different spatial distributions of inclusions but the same percentage of different fractions. The failure at room temperature was modelled.

The specimens were loaded by setting the constant velocity *V_y_* to the elements of the corresponding end surfaces along the Y-axis. Zero external forces were applied to these end surfaces in the horizontal direction to fulfil the boundary condition σ*_xx_* = σ*_xy_* = 0. For quasi-static loading, *V_y_* = 2 mm/s was used, corresponding to the strain rates of 0.4 s^−1^ and 0.25 s^−1^ for compression and tension, respectively. Dynamic loading was performed with the strain rates in the range 5 s^−1^ up to 100 s^−1^. The upper value corresponds to the characteristic strain rate under the dynamic loading of lining by falling scrap.

### 2.3. Homogeneously Deformable Discrete Elements

Computer modelling was carried out using the in-house software MCA 2D Load Test (version 3.0.6.5, ISPMS SB RAS, Tomsk, Russia), which implements the method of homogeneously (simply) deformable discrete elements. In this section, the keystones of this method and the mechanical model of quasi-brittle refractory materials are described.

In comparison with classical DEM, the method of homogeneously deformable discrete elements allows accounting for the stress–strain state of the elements, including their volume strains [39,40,44,45]. It also allows for more accurate implementation of mechanical strength criteria, including the Drucker–Prager criterion. This implementation of DEM is based on the following approximations.

First, the evolution of the ensemble of elements is determined by solving the Newton–Euler system of equations of motion for equivalent balls with central and tangential interaction [37,46].

The second approximation is the interaction of discrete elements through flat faces. For the interacting pair of unstrained elements, the initial value of the area of such a face equals the area of the common face of corresponding polyhedrons, which account for both for the equivalent balls and the “as if filled” voids between them [40]. The introduction of the contact area allows operating with central (normal) and tangential (shear) stresses.

The third approximation is a uniform distribution of strains and stresses in the volume of the element. The averaged values of the stress tensor components in the volume of the element ${\bar{\sigma }}_{\alpha \beta }$ are determined according to [37,39]:

(1) $$\begin{array}{cc} \; & {\bar{\sigma }}_{\alpha \beta }^{i}=\frac{R_{i}}{\Omega_{i}^{0}}\sum_{k=1}^{N_{i}}{\left({\vec{n}}_{ik}\right)}_{\alpha }{\left({\vec{F}}_{ik}\right)}_{\beta }=\frac{R_{i}}{\Omega_{i}^{0}}\sum_{k=1}^{N_{i}}{\left({\vec{n}}_{ik}\right)}_{\alpha }{\left({\vec{F}}_{ik}^{centr}+{\vec{F}}_{ik}^{tang}\right)}_{\beta }=\\ \; & \;\;\;\;\;\;=\frac{R_{i}}{\Omega_{i}^{0}}\sum_{k=1}^{N_{i}}S_{ik}^{0}{\left({\vec{n}}_{ik}\right)}_{\alpha }{\left({\vec{T}}_{ik}\right)}_{\beta }=\frac{R_{i}}{\Omega_{i}^{0}}\sum_{k=1}^{N_{i}}S_{ik}^{0}{\left({\vec{n}}_{ik}\right)}_{\alpha }\left(\sigma_{ik}{\left({\vec{n}}_{ik}\right)}_{\beta }+\tau_{ik}{\left({\vec{t}}_{ik}\right)}_{\beta }\right) \end{array}$$

where *R_i_* is the radius of the equivalent sphere approximating the element *i*, $\Omega_{i}^{0}$ is the volume of unstrained element *i*, $S_{ik}^{0}$ is the contact area in unstrained pair *i–k*, ${\vec{F}}_{ik}$ is the vector of interaction force, ${\vec{F}}_{ik}^{centr}$ and ${\vec{F}}_{ik}^{tang}$ are central and tangential contributions to the total force (${\vec{F}}_{ik}={\vec{F}}_{ik}^{centr}+{\vec{F}}_{ik}^{tang}$), ${\vec{T}}_{ik}$ is the traction vector at the area of contact of elements *i* and *k*, $\sigma_{ik}$ and $\tau_{ik}$ are central and tangential components of *T_ik_* in the pair *i-k*, ${\vec{n}}_{ik}$ is the unit normal vector directed along the line connecting the mass centres of the elements, ${\vec{t}}_{ik}$ is the unit tangent vector directed in the tangential plane, and ${\left(\vec{W}\right)}_{\beta }$ is the projection of some vector $\vec{W}$ onto the β-axis of lab coordinates. The components of the average strain tensor ${\bar{\varepsilon }}_{\alpha \beta }^{i}$ are calculated incrementally with the use of the material’s constitutive law and calculated average stresses [40].

The fourth approximation is that spatial parameters of element–element interaction are divided into the contributions of both elements:

(2) $$\left\{\begin{array}{l} \Delta h_{ik}=\Delta q_{ik}+\Delta q_{ki}=\Delta \varepsilon_{ik}R_{i}+\Delta \varepsilon_{ki}R_{k}\\ \Delta l_{ik}=\Delta \gamma_{ik}R_{i}+\Delta \gamma_{ki}q_{ki} \end{array}\right..$$

Hereinafter, the symbol Δ denotes an increment of the corresponding parameter per time step Δ*t*, *h_ij_* is element–element overlap, *q_ik_* and *q_ki_* are the distances from mass centres of elements *i* and *k* to the centre of interaction area (*q_ik_* + *q_ki_* = *r_ik_*; *r_ik_* is the distance between mass centres of the elements; *q_ik_* = *R_i_* and *q_ki_* = *R_k_* for the unstrained elements), *l_ij_* is the relative shear displacement of elements in the pair *i–k* [37], ε*_ik_* and ε*_ki_* are the central strains of the elements *i* and *k* in the pair *i–k*, γ*_ik_* and γ*_ki_* are the corresponding shear angles of the elements in the pair.

The fifth approximation is that the relation for the central force of element–element interaction takes into account the volumetric part of internal stresses in the interacting elements (mean stresses):

(3) $$\left\{\begin{array}{l} \sigma_{ik}=\sigma_{ik}^{pair}\left(\varepsilon_{ik}\right)+A_{i}{\bar{\sigma }}_{mean}^{i}=\sigma_{ki}=\sigma_{ki}^{pair}\left(\varepsilon_{ki}\right)+A_{k}{\bar{\sigma }}_{mean}^{k}\\ \tau_{ik}=\tau_{ik}^{pair}\left(\gamma_{ik}\right)=\tau_{ki}=\tau_{ki}^{pair}\left(\gamma_{ki}\right) \end{array}\right.,$$

where the index “*pair*” denotes pair-wise function, ${\bar{\sigma }}_{mean}^{i}=\left({\bar{\sigma }}_{xx}^{i}+{\bar{\sigma }}_{yy}^{i}+{\bar{\sigma }}_{zz}^{i}\right)/3$ is the mean stress in the element *i*, and *A_i_* is the material parameter. The tangential interaction is formulated in pair-wise form as in classical DEM formalism. Note that Equation (3) takes into account the necessity to satisfy Newton’s third law (σ*_ik_* = σ*_ki_* and τ*_ik_* = τ*_ki_*).

The last two approximations (Equations (2) and (3)) are the consequences of the deformability of the discrete element and the uniform (homogeneous) distribution of stresses and strains in the volume of the element. Another consequence of the above approximations is that the particular expressions for central and tangential element–element interaction forces (Equation (3)) replicate the expressions for the constitutive relations of the modelled material.

#### 2.3.1. The Linear–Elastic Behaviour of Discrete Elements

The mechanical response of all components of the model is assumed to be isotropic and linear–elastic prior to fracture. The corresponding constitutive equation is Hooke’s law:

(4) Δσαα=2GΔεαα+(1−2GK)ΔσmeanΔταβ=2GΔεαβ,

where α, β = *x*,*y*,*z*; σ_αα_ and ε_αα_ are the diagonal components of stress and strain tensors; τ_αβ_ and ε_αβ_ are the off-diagonal components; $\sigma_{mean}=\left(\sigma_{xx}+\sigma_{yy}+\sigma_{zz}\right)/3$ is the mean stress; *K* is the bulk modulus; and *G* is the shear modulus. The corresponding formulation of the relationships for specific forces σ*_ik_* and τ*_ik_* (response of element *i* to the action of the neighbour *k*) is similar [39,40,44]:

(5) $$\left\{\begin{array}{l} \Delta \sigma_{ik}=2G_{i}\Delta \varepsilon_{ik}+\left(1-\frac{2G_{i}}{K_{i}}\right)\Delta {\bar{\sigma }}_{mean}^{i}\\ \Delta \tau_{ik}=2G_{i}\Delta \gamma_{ik} \end{array}\right.,$$

where *G_i_* and *K_i_* are corresponding elastic moduli for the material of the element *i*. Equations (4) and (5) are written in the hypo-elastic form. Then, Equations (2) and (3) take the following form:

(6) $$\left\{\begin{array}{l} \begin{array}{l} \sigma_{ik}^{cur}=\sigma_{ik}^{pre}+\Delta \sigma_{ik}=\sigma_{ik}^{pre}+2G_{i}\Delta \varepsilon_{ik}+\left(1-\frac{2G_{i}}{K_{i}}\right)\Delta {\bar{\sigma }}_{mean}^{i}=\\ =\sigma_{ki}^{cur}=\sigma_{ki}^{pre}+\Delta \sigma_{ki}=\sigma_{ki}^{pre}+2G_{k}\Delta \varepsilon_{ki}+\left(1-\frac{2G_{k}}{K_{k}}\right)\Delta {\bar{\sigma }}_{mean}^{k} \end{array}\\ \Delta h_{ik}=\Delta \varepsilon_{ik}R_{i}+\Delta \varepsilon_{ki}R_{k} \end{array}\right.,$$

(7) $$\left\{\begin{array}{l} \tau_{ik}^{cur}=\tau_{ik}^{pre}+\Delta \tau_{ik}=\tau_{ik}^{pre}+2G_{i}\Delta \gamma_{ik}=\tau_{ki}^{cur}=\tau_{ki}^{pre}+\Delta \tau_{ki}=\tau_{ki}^{pre}+2G_{k}\Delta \gamma_{ki}\\ \Delta l_{ik}=\Delta \gamma_{ik}R_{i}+\Delta \gamma_{ki}R_{k} \end{array}\right..$$

Here, the upper indexes “*cur*” and “*pre*” denote values of specific forces at the current and previous time steps of integrating the motion equations.

A pair of discrete elements can be in a bound or unbound state. The connected pair of elements resists tension and compression as well as shear. An unbound pair resists only compression, and the value of shear resistance is limited from above by the value of the dry friction force: $\left|\tau_{ik}\right|\leq \mu \left|\sigma_{ik}\right|$, where μ is the local value of the friction coefficient [37].

#### 2.3.2. Quasi-Static Model of Local Fracture

Local failure is modelled as a bonded-to-unbonded transition. We considered two alternative failure criteria, namely the Drucker–Prager criterion and the combined Drucker–Prager–Rankine criterion.

The Drucker–Prager criterion takes into account the dependence of shear strength on local pressure, which is essential for all brittle and quasi-brittle materials [47,48]:

(8a) $$\sigma_{fract}=1.5\left(\beta -1\right)\sigma_{mean}+0.5\left(\beta +1\right)\sigma_{eq}=\sigma_{c}$$

where β = σ*_c_*/σ*_t_*, σ*_c_*, and σ*_t_* are the values of the uniaxial tensile strength (UTS) and uniaxial compressive strength (UCS) for the component or the interface.

It is known that the Drucker–Prager failure criterion may overestimate the failure stress in the parametric area of tensile volumetric stresses. Thus, this criterion is often supplemented by the Rankine condition:

(8b) $$\sigma_{1}=\sigma_{t},$$

where σ_1_ is maximum principal stress. Rankine criterion limits the main tensile stress to the tensile cut-off.

The model of local fracture includes not only the condition for bonded-to-unbonded transition (fracture criterion) but also the separation of the surfaces. In this way, when the failure criterion is met, the cohesion/adhesion of the elements is gradually lost. Loss of cohesion/adhesion is regarded as a decrease in the proportion of the bonded part of the contact surface over time. It is controlled by the dimensionless parameter *k_bond_*, which is the ratio of the bonded part of the contact surface to the total value of the contact surface (0 ≤ *k_bond_* ≤ 1). Two limiting values are *k_bond_* = 0 (totally unbonded pair) and *k_bond_* = 1 (totally bonded pair). The bonded part of the contact area in the *i–k* pair is $S_{bond}^{ik}=S_{ik}k_{bond}^{ik}$.

For the elastic–brittle large grains, the model of the dynamic separation of the surfaces at a constant rate was applied:

(9) $$\frac{dk_{bond}}{dt}=-\upsilon_{s}<0.$$

Here, *t* is time, and υ*_s_* is the rate of bond breaking. This model assumes that not only the beginning but also the continuation of the separation of surfaces at time *t* takes place when the fracture criterion is met at a given time. The dynamic surface separation model is based on describing a brittle bond break as the unstable crack propagation along the contact surface. For convenience, we use the velocity of propagated crack *V_s_* = υ*_s_R*. Here, *R* is the element radius. The parameter *V_s_* is the input parameter of the model. The value of this parameter is physically limited by Rayleigh wave velocity *V_R_*.

For the porous matrix, a surface separation model is based on the approximation of stable crack growth. The surface separation rate is variable, depending on the dynamics of deformation of the pair. The current rate of separation of surfaces is proportional to the sum of the current values of the rate of expansion and the rate of relative shear of the elements of the pair under consideration:

(10) $$\frac{dk_{bond}}{dt}=-\left(\frac{1}{\varepsilon_{\max}}\frac{d\varepsilon_{pair}}{dt}+\frac{1}{\gamma_{\max}}\frac{d\gamma_{pair}}{dt}\right)<0.$$

Here, ε_max_ and γ_max_ are dimensionless input parameters of the model; *d*ε*_pair_* and *d*γ*_pair_* are changes in the central strain and the shear strain in the couple of elements during the time *dt*. For the pair of elements *i–k*, these changes are defined as follows:

(11) $$\begin{array}{c} d\varepsilon_{pair}^{ik}=\left\{\begin{array}{l} dh_{ik}/\left(R_{i}+R_{k}\right),\;\;\;if\;dh_{ik}>0\\ 0,\;\;\;\;\;\;\;\;\;\;\;\;\;\;\;\;\;\;\;\;if\;dh_{ik}\leq 0 \end{array}\right.\\ d\gamma_{pair}^{ik}=\left\{\begin{array}{l} d\left|l_{ik}\right|/\left(R_{i}+R_{k}\right),\;\;\;if\;d\left|l_{ik}\right|>0\\ 0,\;\;\;\;\;\;\;\;\;\;\;\;\;\;\;\;\;\;\;\;\;if\;d\left|l_{ik}\right|\leq 0 \end{array}\right. \end{array}.$$

The physical meaning of ε_max_ is the normalized change of distance between the elements when under the condition of uniaxial tension, the cohesion is fully lost. The physical meaning of γ_max_ is analogous and relates to the condition of pure shear.

The continuation of the separation of surfaces at time *t* is applied when the fracture criterion is met at a given time. The described “deformation” model is in many respects similar to well-known non-potential cohesive zone models, which are used in finite element modelling. The validity of using such a model for a porous matrix is justified by the fact that highly porous materials often exhibit a rough crack trajectory.

#### 2.3.3. Modelling of the Grain–Matrix Interfaces

The interface zones connecting the matrix and inclusions were modelled in the approximation of an infinitely small interface thickness. The approximation is valid because the matrix and the rims of the grains have similar chemical and mineralogical compositions. Zones of apparent increase of porosity at the interface are not properly quantified. Thus, the only characteristics of the interfaces are the parameters of the Drucker–Prager strength criterion and the model of adhesion loss. The model of stable crack growth (Equations (10) and (11)) was used.

#### 2.3.4. Dynamic Formulation of Fracture Criterion

The classical fracture criteria, including Drucker–Prager failure criterion (Equation (8a)), are quasi-static, since they do not take into account the finite time of damage and crack formation. This can lead to a significant underestimation of the dynamic strength, which is seen under the loading strains of 10 s^−1^ and higher [49]. The general dynamic formulation of the Drucker–Prager failure criterion was used:

(12a) $$\sigma_{fract}\left(t\right)=1.5\left(\beta_{dyn}-1\right)\sigma_{mean}\left(t\right)+0.5\left(\beta_{dyn}+1\right)\sigma_{eq}\left(t\right)=\sigma_{c}^{dyn}\left(t-t_{0}\right).$$

Here, $\beta_{dyn}=\sigma_{c}^{dyn}\left(t-t_{0}\right)/\sigma_{t}^{dyn}\left(t-t_{0}\right)$; $\sigma_{c}^{dyn}\left(t-t_{0}\right)$ and $\sigma_{t}^{dyn}\left(t-t_{0}\right)$ are dynamic values of compressive and tensile strength, respectively, *t*_0_ is the starting time of incubation of fracture when the parameter σ*_fract_* reaches the static strength $\sigma_{c}=\sigma_{c}^{st}$; *T_fract_* = *t* − *t*_0_ is the time parameter of local failure. The dynamic Rankine condition is defined as follows:

(12b) $$\sigma_{1}\left(t\right)=\sigma_{t}^{dyn}\left(t-t_{0}\right).$$

The input functions $\sigma_{c}^{dyn}\left(T_{fract}\right)$ and $\sigma_{t}^{dyn}\left(T_{fract}\right)$ can be determined from standard laboratory dynamic tests [49]. Those functions are material properties.

It is known that despite significant differences in the absolute values of the dynamic strength of various brittle materials, the dependence of their normalized strength on the strain rate can be approximated with acceptable accuracy by a single curve [50,51,52,53]. There is the following set of unifying dependences [49]:

(13) $$\frac{\sigma_{c}^{dyn}(T_{fract})}{\sigma_{c}^{st}}=\frac{1}{f_{scale}}\left\{\begin{array}{l} 1-0.37\ln\left(\frac{T_{fract}}{F_{c}T_{1}^{}}-0.01\right)\;\;\;\;\;\;\;\;at\;T_{fract}^{}\leq F_{c}\cdot 3.762\cdot 10^{-4}s\\ 1-0.83\ln\left(\frac{T_{fract}}{F_{c}T_{2}^{}}-0.0129\right)\;\;\;\;\;at\;T_{fract}^{}>F_{c}\cdot 3.762\cdot 10^{-4}s \end{array}\right.,$$

(14) $$\frac{\sigma_{t}^{dyn}(T_{fract})}{\sigma_{t}^{st}}=\frac{1}{f_{scale}}\left\{\begin{array}{l} 1-1.66\ln\left(\frac{T_{fract}}{F_{t}T_{3}^{}}-0.238\right)\;\;\;\;\;at\;T_{fract}\leq F_{t}\cdot 4.623\cdot 10^{-5}s\\ 1-0.19\ln\left(\frac{T_{fract}}{F_{t}T_{4}^{}}-0.164\right)\;\;\;\;\;at\;T_{fract}>F_{t}\cdot 4.623\cdot 10^{-5}s \end{array}\right..$$

Here, *T*_1_ = 10^−3^ s, *T*_2_ = 1.55 × 10^−2^ s, *T*_3_ = 5.25 × 10^−5^ s, and *T*_4_ = 2.5 × 10^−4^ s. Nondimensional parameters $F_{c}=\frac{\sigma_{c}^{st}}{E}/\frac{{\left(\sigma_{c}^{st}\right)}^{ref}}{E^{ref}}$ and $F_{t}=\frac{\sigma_{t}^{st}}{E}/\frac{{\left(\sigma_{t}^{st}\right)}^{ref}}{E^{ref}}$ characterize the ratios between Young’s modulus (*E*), the static strengths (σ*_c_^st^* and σ*_t_^st^*) of the studied material, and the properties of the known reference material. Here, the expressions were derived using the properties of consolidated sandstone (*E^ref^* = 16 GPa, (σ*_c_^st^*)*^ref^* = 70 MPa, (σ*_t_^st^*)*^ref^* = 10 MPa). The parameter *f_scale_* is a dimensionless scale factor that characterizes the ratio of the scale of macroscopic samples (*H_macro_* ≈10^−2^ m) to the considered scale of fracture. For particle-reinforced composite, as the modelled refractory material, such parameter is the characteristic distance between the centres of the modelled large grains $H_{heter}=H_{meso}/\sqrt{N_{inc}}$, where *H_meso_* is the sample size, and *N_inc_* is the number of inclusions in the model composite sample. For the modelled refractory, *f_scale_* is estimated as the ratio *f_scale_*=*H_macro_*/*H_heter_* ≈ 25. For grain–matrix interfaces, the Young’s modulus used in Equations (13) and (14) was set as equal to that of the matrix, as it is the softest constituent.

### 2.4. Model Parameters

The properties of the microstructure’s constituents were estimated from the experimental data (Table 1). The grains’ density was made equal to the true density of the material, while the density of the matrix was calculated from the total porosity, apparent, and true density—see Section 2.1. In the calculation, it was assumed that all the porosity of the material is localised in the matrix. The Young´s modulus of the grains was set equal to the dynamic modulus of cristobalite [54]. The Young’s modulus of the matrix was calculated using Counto’s equation for the bi-phasal materials. The reverse analysis was performed for the known parameter of the grains, aiming for the total Young’s modulus of 6 GPa. The latter value was chosen regarding the fact that the Young’s modulus found in bending and compressive tests was 4–6 GPa and 6–7 GPa, respectively. As the first approximation, the strength of the grains was estimated by extrapolating the correlations of the sample strength and portion of the grain through the grain fracture trajectory [12]. The tensile strengths of the matrix and interface between the large grain and matrix were obtained by scaling those to the strength of the large grains. For that, Knoop hardness tests (Shimadzu HMV-2, Kyoto, Japan) performed on different constituents of the microstructure of the modelled material were used [12]. The ratio of the strength between the different constituents was equal to the differences between average Knoop hardness values for the respective constituents.

The ratio between the compressive and tensile strengths of the constituents was calculated from the relationship of $\sigma_{c}^{st}/\sigma_{t}^{st}=\left(\sqrt{3}+\tan\varphi \right)/\left(\sqrt{3}-\tan\varphi \right)$ [55]. The experimental data on refractories [56,57] reveal that the angle of friction φ can vary in the wide range of, at least, between 35–45° and 60–70°. For the given range, the ratio can vary between 2 and 9. Regarding these data, the initial assumption for the grains, matrix, and interfaces was $\sigma_{c}^{st}/\sigma_{t}^{st}$ = 4 (Table 1).

The following parameter was used to model the separation of the surfaces upon failure (Table 2). For the grains, *V_s_* = 0.5*V_R_*, which is representative of the dense, brittle materials. For the matrix and interfaces, the initial values of ε_max_ and γ_max_ are the average values of the post-peak strain seen in compression and bending tests (Figure 1b,c). In general, those parameters are 10% of the ratio $\sigma_{t}^{st}/E$.

The value of the local coefficient of friction μ was set equal to 0.1. It was seen that varying the value of μ in the range from 0 to 0.3 does not lead to a significant change in the fracture pattern and mechanical characteristics of the samples under unconfined uniaxial loading.

## 3. Results and Discussion

### 3.1. Validation of the Model and the Adjustment of the Local Properties

The reference characteristics that were utilised in assessing the accuracy of the mechanical model were the quasi-static values of the ultimate tensile (UTS) and compressive (UCS) strengths, post-peak behaviour, and parameters of the crack trajectory. The procedure for evaluating and refining model parameters described below is a sequence of logically connected stages.

#### 3.1.1. Quasi-Static Fracture Criterion

**Figure 3:**
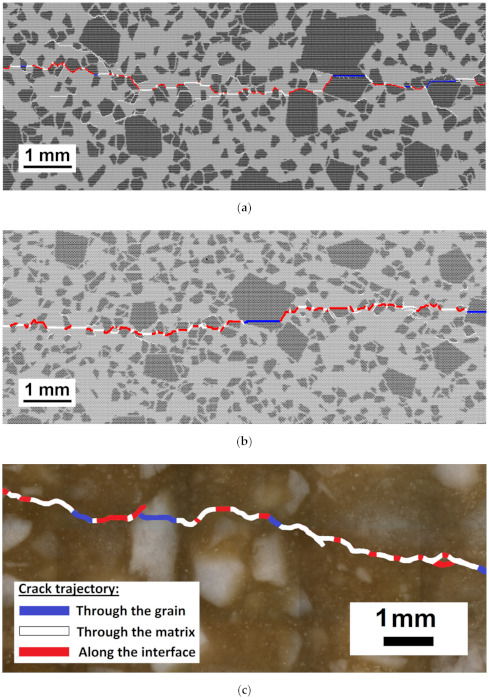
Examples of the fracture pattern obtained with Drucker–Prager criterion (**a**), combined Drucker–Prager–Rankine criterion (**b**), and a tensile crack formed in a sample of silica refractory under monotonic wedge splitting loading (**c**). The thick curved line in (**c**) shows the crack trajectory. White, blue, and red portions of the crack lines indicate crack propagation through matrices, through grains, and along interfaces, respectively. The width of each image represents 1 cm of the sample.

First, an examination of the alternative failure criteria and values of their parameters (Table 1) was carried out. Figure 3 illustrates a typical example of a fracture pattern under uniaxial tension for the criteria (8a) and (8b). The fracture occurs by the formation of a transverse main crack in the middle of the specimen. For the model with the Drucker–Prager criterion, the main crack trajectory has many branches and small internal cracks on both sides of the main crack’s line (Figure 3a). This type of fracture under tensile conditions is more likely to occur in loosely bound materials [58] but is not typical for silica refractory. Experimental data [12] indicate that tensile cracks in silica refractory are localised. The combined Drucker–Prager–Rankine criterion provides the required localised fracture pattern (Figure 3b), which is qualitatively appropriate with the experimental data (Figure 3c). The Rankine criterion had little effect on the fracture pattern and strength in the compressive models. The results displayed in the following sections are obtained using the combined Drucker–Prager–Rankine criterion.

#### 3.1.2. Strength of Constituents

**Figure 4:**
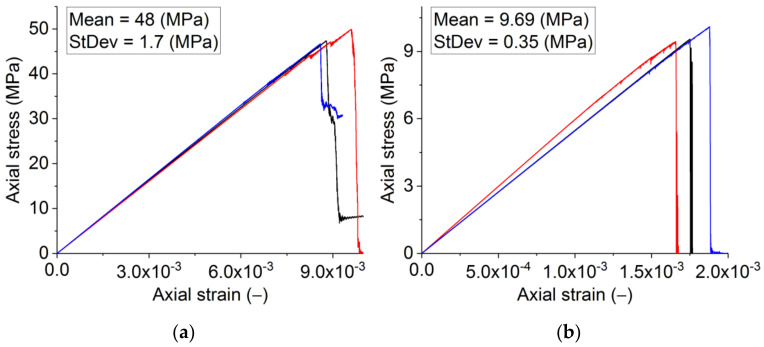
Numerically obtained uniaxial compression (**a**) and tension (**b**) diagrams with the use of the final strength parameters for mesoscopic structural elements. Diagrams are shown for the three statistically equivalent samples.

Under tension and compression, the modelled material had a Young’s modulus of 5.5 GPa (Figure 4). Thus, the selected elastic characteristics of the constituents (Table 1) produce an acceptable representation of Young’s modulus, as seen in the lab tests. However, the obtained strength was an order of magnitude lower than the experimental values. Furthermore, the numerically obtained strength ratio UCS/UTS ≈ 3.8 is lower than the experimental value of approximately 5 (Figure 1).

To match the laboratory data of macroscopic strength, the strength of large grains, matrix, and interfaces was increased. It was assumed that although the strengths of the constituents were underestimated (Table 1), their relative values are correct—this assumption is summarised in Table 3. Note that this implicitly assumes the same ratio σ*_c_*/σ*_t_* for all structural elements.

The determination of the values of the local strength of structural elements was executed in two stages. In the first stage, we varied the σ_c_/σ_t_ ratio for the structural elements to obtain the required UCS/UTS. We increased the values of σ*_c_* of the structural elements while maintaining constant values of σ*_t_*. The required ratio of macroscopic strengths UCS/UTS = 5 is achieved at σ*_c_*/σ*_t_* = 10. In the second stage, we jointly increased σ*_c_* and σ*_t_* for structural elements while keeping their σ*_c_*/σ*_t_* ratio unchanged, thus rendering it possible to achieve the required values of uniaxial compressive and tensile strength of the samples (UCS ≈ 48 MPa, UTS ≈ 9.7 MPa, UCS/UTS = 5). The stress–strain curves after the final tuning are presented in Figure 4a,b. Corresponding values of local strength are shown in Table 4. One should note that the values of σ*_c_* and σ*_t_* (UCS and UTS) obtained for the grains are similar to those of dense silica [59]. However, unlike in the model, the real stress–strain curves exhibit some non-linearity at the peak. This effect will be addressed further in Section 3.1.4.

The analysis of the tensile stress–strain curves indicated the fracture energy of ≈90 N/m. The stress–strain curve demonstrates the brittle failure. For quasi-brittle materials, this was observed when the elastic energy released upon the crack formation exceeds the energy to be consumed by the forming crack. This balance is influenced not only by the material properties but also by the shape of the loaded body [60]. For the same reason, brittle failure is observed on the laboratory three-point bending curves (Figure 1c). The energy from the stress–strain curves showing brittle failure indicates the elastic energy released, which exceeds the fracture energy. Therefore, it is not surprising that the fracture energy obtained by us exceeds the fracture energy of 60 N/m obtained for this material in the stable regime of crack propagation enabled by the wedge-splitting test [42].

Here, it should be noted that the compared virtual and real samples have different shapes and boundary conditions. For instance, due to a higher height to width ratio, the real compressive sample is expected to have somewhat lower strength and more brittle failure. However, the friction between the loading plates and sample, which is absent in the model, can reduce these effects. The lab data of purely uniaxial tensile and compressive tests could be used to further improve the model.

**Figure 5:**
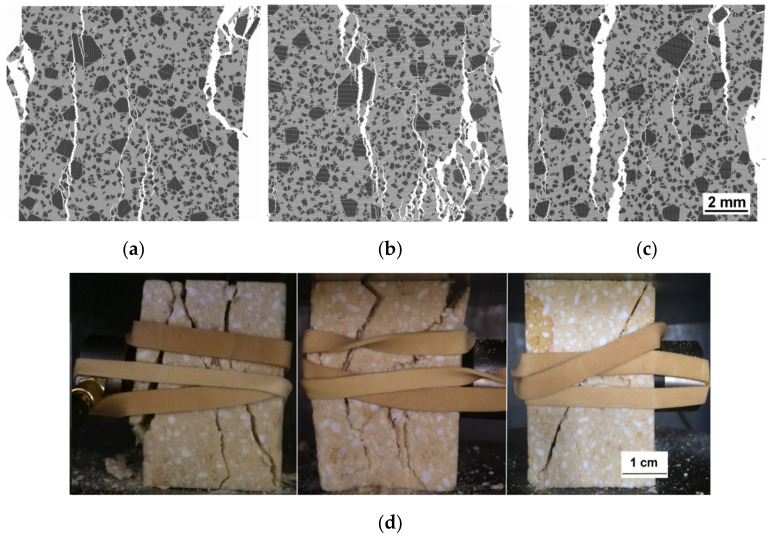
Crack patterns for uniaxial compression tests (**a**–**c**) models with final values of local strength parameters (1 × 1 cm^2^) (**a**–**c**), typical crack patterns in the real samples (**d**).

**Figure 6:**
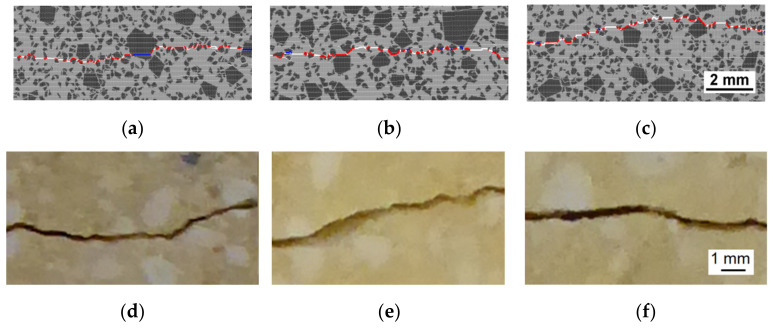
Crack patterns of the model samples under uni-axial tension (**a**–**c**) and typical crack patterns in the real samples under three-point bending (**d**–**f**). White, blue, and red portions of the crack lines in (**a**–**c**) indicate crack propagation through matrices, through grains, and along interfaces, respectively. The width of each image represents 1 cm of the sample. See also Supplementary Materials with full-size (**a**–**c**).

The fracture patterns for the “statistically equivalent” models with final values of local strength parameters (Table 4) are compared with those registered in the refractory samples. For uniaxial compression, one can see that all DEM samples display similar fracture patterns, namely the vertical main cracks split the sample into several large parts (Figure 5a–c). The trajectories of tensile cracks have a localised nature (Figure 6a–c). The modelled failure is rather similar to that in real samples (Figure 5d and Figure 6d–f).

#### 3.1.3. Properties of the Interface

**Figure 7:**
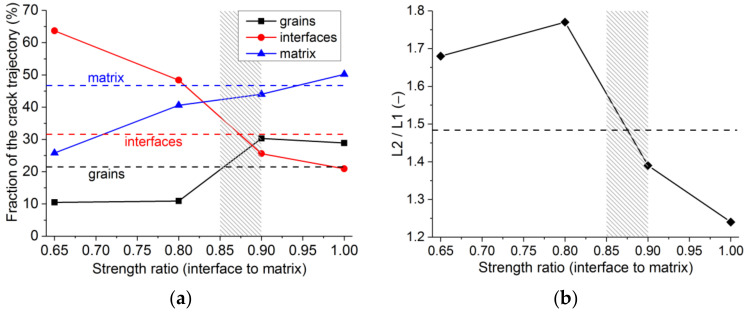
Comparison of the virtual and real crack trajectories for crack portions through different constituents of the model samples (**a**) and the crack non-linearity parameter L2/L1 (**b**). Horizontal dashed lines denote experimental values for silica refractory [12]. The rectangular shaded area marks the interval where the model is considered to agree with the experiment.

To validate the initial estimate of the interface strength (Table 4), the analysis of the crack trajectory was performed (Figure 7). Calculations for the interface’s strength between 65% and 100% of the strength of the matrix were conducted. The initially set ratio of the matrix strength to interface strength of 1.5 corresponds to the value of 66.7%. In all cases, σ*_c_* and σ*_t_* for interfaces were varied synchronously, keeping the ratio σ*_c_*/σ*_t_* = 10 as constant. In modelled crack trajectories, the portions of the crack propagating through the interface, large grains, and matrix were measured. Additionally, the ratio of the crack length (L2) to its projection on the plane parallel to the direction of the crack propagation (L1) was calculated. The latter ratio quantified the crack’s non-linearity. Averaged results for the three model samples were weighed against the experimental crack propagation analysis for the same grade of silica refractories, for which the experimental cracks were obtained in the wedge-splitting test [12]. In the fractographic analysis, six crack trajectories of three wedge-splitting tests were analysed. Per sample, two polished sections exposing cracks at different depths of the sample were used.

For the models with low interface strength, the portion of the crack trajectory through the interface approaches 0.7 (Figure 7a). The crack jumps from the interface of one large grain to another, producing a trajectory of high non-linearity (L2/L1). For the models where the interface’s strength approaches that of the matrix, the preferred crack propagation route is through the matrix. When a crack meets a grain, it splits it, causing the non-linearity of the crack to become lower (Figure 7b). The best agreement between the parameters of the modelled and experimental crack trajectory is achieved for the interface strength which is 85–90% of the matrix strength.

An increase of the interface strength from 65% to 100% produces the average increase of UTS of ≈10% (from 9.7 to 10.7 MPa) and UCS of ≈25% (from 48 to 59.7 MPa). The strength ratio increases approximately by 10% (UCS/UTS ≈ 5.5). In any case, the strength values and their ratio are in the characteristic range these parameters have in the real silica refractory (Figure 1b,c).

The observations of different crack trajectories and almost identical strength values, which is observed here for samples with different interface strengths, is in agreement with lab test results [12]. There, different participation of the interface failure was detected for two groups of samples tested under monotonic and cyclic wedge splitting. However, the average strength values for both groups were similar. Apart from the global strength, the interface strength is to influence the fracture energy. The correlation of the fracture energy and the interface strength is to have the maximum. DEM modelling may be used to find the optimum enabling best combination of the global strength and the fracture energy.

#### 3.1.4. Influence of Finite Time of Fracture Incubation

Obtained curves (Figure 4), especially for compression, exhibit unrealistically brittle failure. To explore the possibility of controlling the length of the sample softening section of the stress–strain curve under compression, the parameters ε_max_ and γ_max_ were varied. The values of these parameters decreased and increased within five times relative to the “basic” values (Table 2). As above, we assumed ε_max_ = γ_max_. In all cases, the values were the same for the matrix and interfaces. The variation of the parameters had a limited effect on the softening. Particularly, the parameter ε_max_ does influence the length of the softening slope. However, this effect is limited to crack formation. The failure remains brittle.

Further adaptation of the model was to account for the finite time of incubation of damage, i.e., to use the dynamic model with combined criteria (12)–(14). The observed brittle compressive failure of the model samples is believed to be a consequence of the strain rate (≈10^−1^ s^−1^) far beyond the typical quasi-static range (10^−3^–10^−5^ s^−1^) in experimental studies of refractory samples. The relatively large strain rate was used in the simulation to reduce the analysis time. At such characteristic values of loading rates (≥10^−1^ s^−1^), elastic waves from emerging damages do not have time to completely dissipate before new damages appear and thereby affect the conditions of new damage formation.

To accurately simulate the fracture of the sample at strain rates ≈ 10^−1^ s^−1^, the calculations were implemented using the dynamic model of fracture. The final quasi-static mechanical characteristics of the constituents (Table 4) were used in the dynamic model. The static strength of the interface was set as 90% of the strength of the matrix (σ*_c_* = 390 MPa, σ*_t_* = 39 MPa).

**Figure 8:**
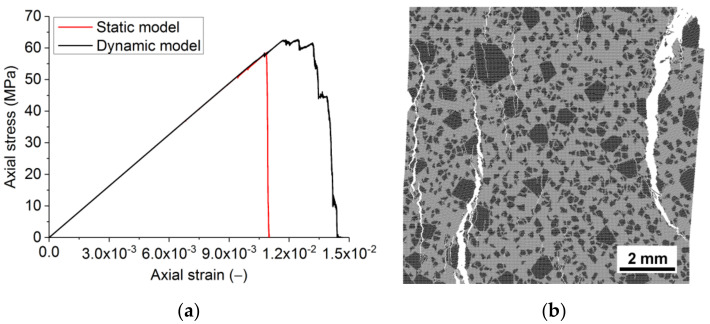
Compression diagrams obtained using the static and dynamic fracture models (**a**) and the broken sample in the dynamic fracture model (**b**). Strain rate 4 × 10^−1^ s^−1^.

**Figure 9:**
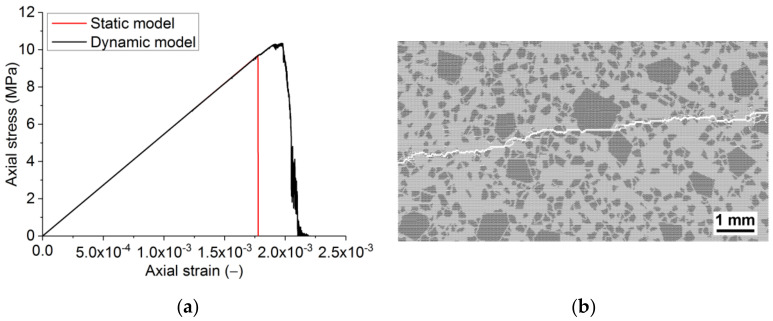
Uniaxial tension diagrams obtained using the static and dynamic fracture models (**a**) and the broken sample in the dynamic fracture model (**b**). Strain rate 2.5 × 10^−1^ s^−1^.

The dynamic model captures the dynamic hardening of materials even at the considered relatively low strain rates (≈10^−1^ s^−1^). Consequently, as a result, the strength of the sample is higher than in the model with static formulation (Figure 8 and Figure 9). The difference is ≈7% and 5–6% for compressive and tensile strengths, respectively. The fracture of the model becomes less brittle. The quasi-brittle nature of the fracture seen under compression (Figure 8) is due to the gradual formation of mesoscopic internal cracks and their coalescence into macrocracks. The length of the softening section for compression and tension is ≈1.5 × 10^−3^ and ≈1.2 × 10^−4^, respectively. The value for compression quantitatively agrees with the data for samples of the real material. One can note that the ratio of the lengths of the softening sections for tension and compression corresponds to that of macroscopic strengths with a proportionality coefficient of ≈2.

### 3.2. Dynamic Mechanical Behaviour

Traditionally, two characteristic strain rate intervals are distinguished: “low” ($\dot{\varepsilon }$ < 10^−3^ s^−1^) and “large” strain rates ($\dot{\varepsilon }$ > 10^0^ s^−1^). In the first interval, the majority of brittle solids can be roughly assumed to be strain rate insensitive [61,62,63]. In the second, the increase of the strain rate causes the non-linear increase of strength. For concrete, for example, the dynamic increase factor (DIF) approaches 2 at $\dot{\varepsilon }$ ≈ 10^2^ s^−1^. DIF is the normalised increase of strength due to the increase of the strain rate from quasi-static to a given dynamic value. From the three-point bending and wedge splitting tests reported for this material [12], strain rates typical for mechanical tests on refractories can be estimated as 3–7 × 10^−5^ s^−1^. Regarding the fact that the height from which the scrap and other solid particles are loaded may be as high as 5–7 m, the velocity at the impact on the face of the refractory lining will exceed the loading velocity in the lab tests by many orders of magnitude. By scaling the velocities of the falling scrap and loading piston of the lab experiments [12], one can estimate the maximal strain rates experienced by refractories as 50–150 s^−1^. Similar magnitudes of strain rates are expected from the impacts of the hydraulic hammer during the repair of the lining.

**Figure 10:**
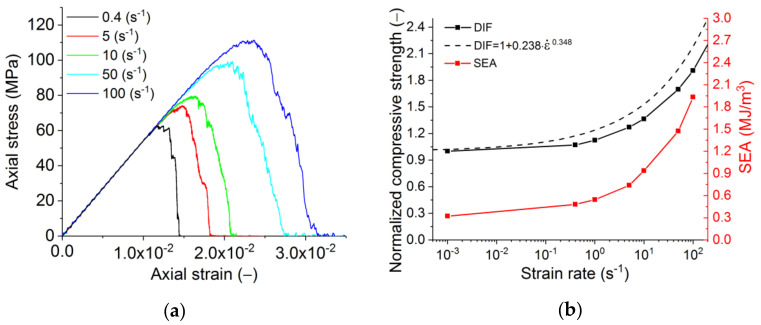
Results for dynamic compressive loading: (**a**) stress–strain diagrams, (**b**) effect of the strain rate on normalized strength and specific energy absorption (SEA). The dashed line is the averaging analytical approximation of experimental data for various brittle and quasi-brittle materials [49].

Dynamic modelling was performed with the material properties, as presented in Table 4, and the interface strength of 90% of the matrix strength. From the analysis, one can observe a non-linear increase of the strength with the increasing rate of loading (Figure 10), which is typical for the vast majority of materials [50,51,52,53]. It should also be noted that the slope of the softening section becomes flatter at high strain rates. This is due to the fact that at high strain rates, the fracture time of the sample becomes comparable to the loading time, as a result of which there is a constant “additional load” of the breaking sample even after the loss of stability.

The energy absorption (or fracture energy) is also an important characteristic of the material. In the simplest case, specific energy absorption (SEA) can be estimated as the area under the loading diagram (σ–ε). In this paper, SEA is determined by the area under the loading curve. The red line in Figure 10b illustrates the change in SEA with an increasing loading rate. Its strain-rate dependence is a non-linear (power-law) function, and SEA’s absolute value in the considered range of strain rates increases by an order of magnitude owing to distributed cracking. To translate it to more conventional means for refractories units of N/m (specific fracture energy), the reported values of SEA should be multiplied by the height of the sample. One should note that this approach neglects the non-linearity of the crack (and multiple cracking under dynamic loading). However, the fracture energy measured for refractories in lab tests also neglects the crack waviness.

For reference, one can consider the SEA of civil engineering concrete measured at 100 s^−1^ in a split Hopkinson pressure bar set-up. SEA was found to be about 1 MJ/m^3^ [64]. The material has a quasi-static compressive strength of 50 MPa. The SEA’s order of magnitude agrees with our modelling results.

**Figure 11:**
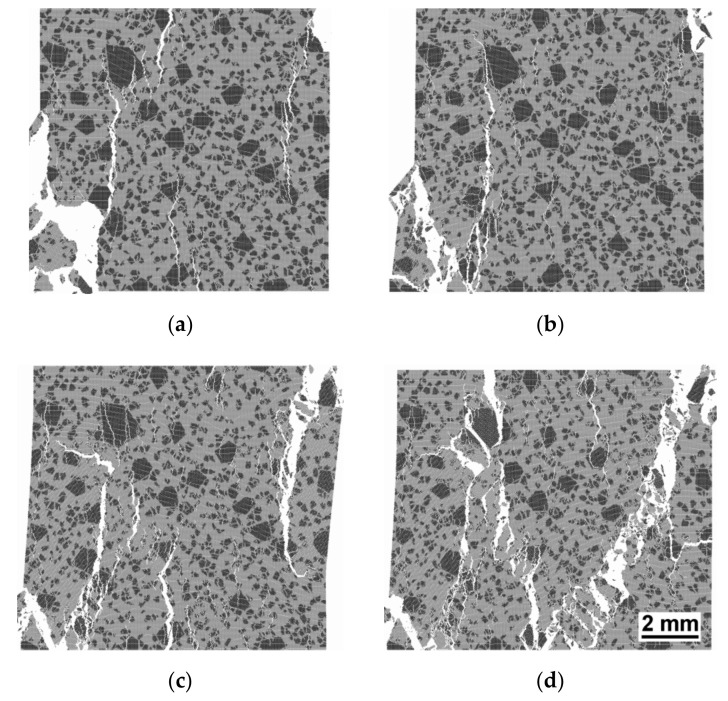
Fracture patterns under dynamic compression for the strain rates of 5 s^−1^ (**a**), 10 s^−1^ (**b**), 50 s^−1^ (**c**), and 100 s^−1^ (**d**).

Multiple increases in SEA and fracture energy at high strain rates are reflected in the fracture pattern (Figure 11). Increased fragmentation of the sample is seen for the increasing rate of loading. This is a typical regularity of the change in fracture pattern for the majority of brittle and quasi-brittle materials under dynamic impact [65].

**Figure 12:**
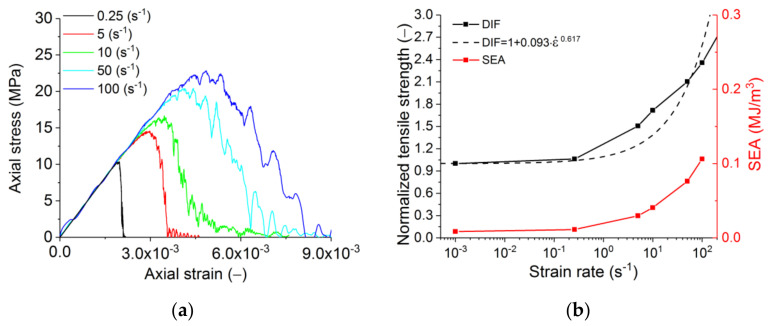
Results for dynamic tensile loading: (**a**) stress–strain diagrams, (**b**) effect of the strain rate on normalised strength and specific energy absorption (SEA). The dashed line is the analytical approximation reported elsewhere [49].

The predicted compression and tension strength values broadly agree with the averaged experimental data for brittle materials [50,51,52,53] (Figure 10b and Figure 12b). In the figures, the latter is shown as the dashed line. The deviations lie within the range of the strength scatter for various brittle and quasi-brittle materials. For refractories, the typical values for the properties scatter are 10–20% of the average value. The deviation is higher for the tensile failure than for compressive failure, especially if one considers the change of slope in the experimental and numerical curves. This feature of the tensile behaviour may indicate that with an increase in the strain rate, the governing mechanism of fracture changes from separation to a mixed mechanism including shear.

Note that the presented averaged dependence was employed to determine the fracture time of the components and parameters of relationships (13) and (14). The differences between the dynamic strength curve of the refractory under consideration and averaged curve are obviously associated with the influence of the internal structure’s features (size and shape of grains, the characteristic distance between them, interfaces, etc.).

**Figure 13:**
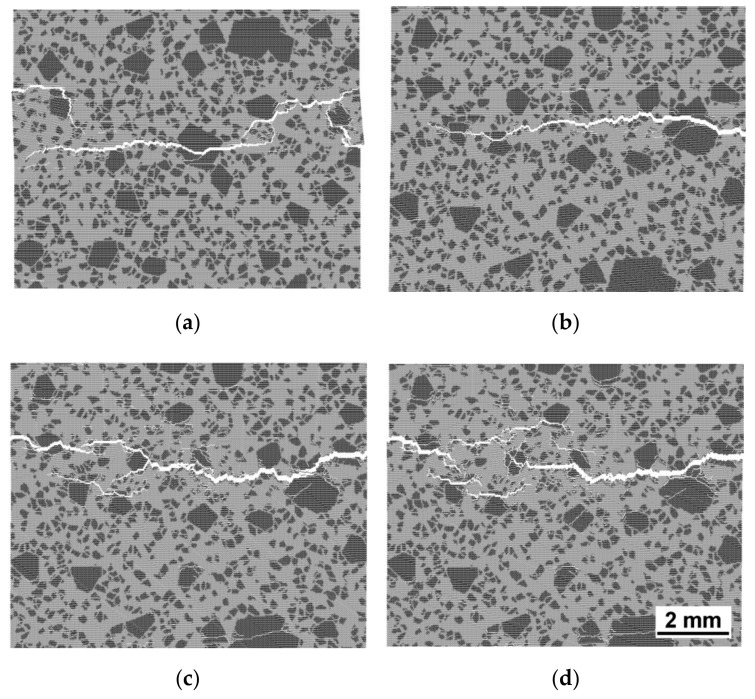
Fracture patterns under dynamic tension for the strain rates of 5 s^−1^ (**a**), 10 s^−1^ (**b**), 50 s^−1^ (**c**), and 100 s^−1^ (**d**).

For dynamic tensile tests, one can perceive that the increasing loading rate produces more developed strain softening (Figure 12a) on account of the transition from the extremely localised fracture at low strain rates to the formation of a system of mesoscopic cracks (Figure 13). The fracture under dynamic loading is characterised by the presence of several centres of growth of transverse mesoscopic cracks (the initial stage of fracture), following which these initial cracks are combined into one main fracture. Due to several fracture sites, the main crack under dynamic loading is much more tortuous than under quasi-static loading.

**Figure 14:**
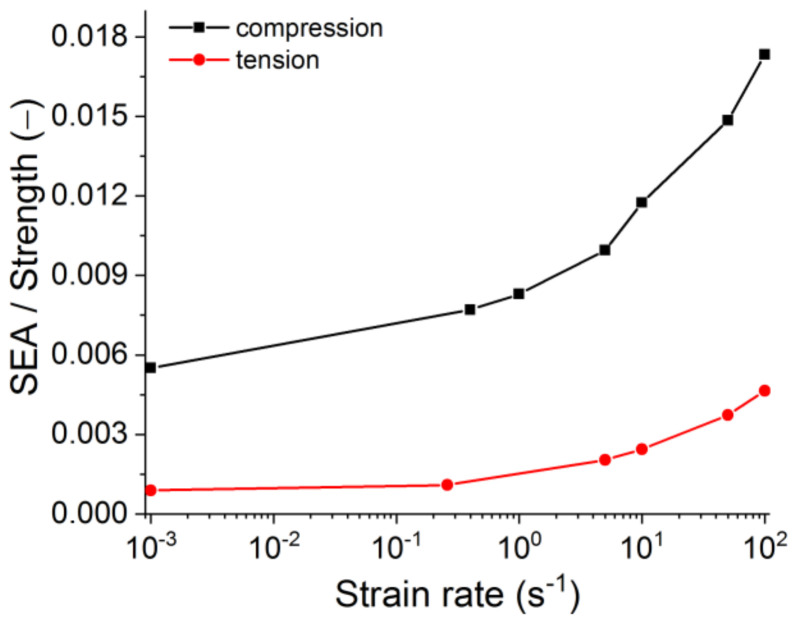
The dependence of the ratio of specific energy absorption to strength on the strain rate.

For refractories, the critical material property is the brittleness at failure. One of the indexes of this property is the ratio of fracture energy to strength: the lower the ratio, the higher the brittleness. With increasing strain rate, the refractory’s strength is identified to vary by approximately two times. For the same interval of strain rates, the fracture energy is identified to vary by ten times. Therefore, increasing of the loading rate causes the decrease of brittleness (Figure 14). As the fracture energy increases, the decreased brittleness should result from a more distributed cracking.

**Figure 15:**
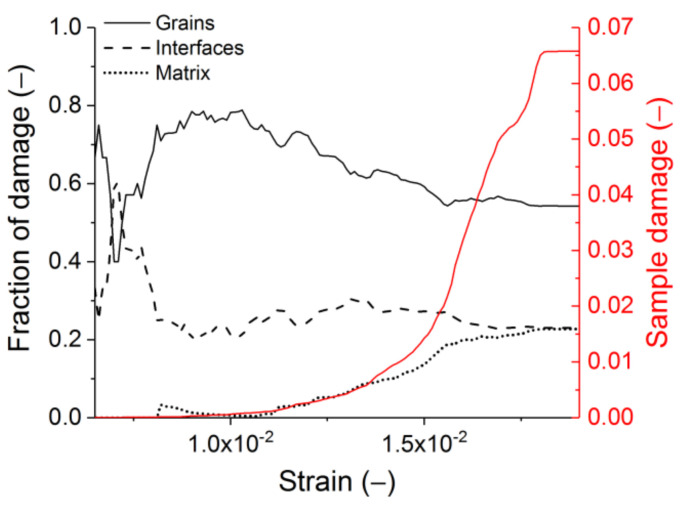
Contributions of constituents to the sample damage accumulation during dynamic compressive loading. The strain rate is 5 s^−1^. The red curve is the absolute sample damage.

Regarding the complex nature of the dynamic failure, the damage development in the process of loading has been analysed. The sample damage was characterised by the number of broken bonds in pairs of discrete elements normalised to the initial number of bonds in the sample. The contribution of each constituent to the sample damage (fraction of damage) was characterised by the ratio of the number of broken bonds in this constituent to the total number of broken bonds in the sample. Figure 15 illustrates how the constituents (grains, matrix, and interfaces) contribute to the damage accumulation in the sample under dynamic loading. The damage starts in the interfaces and grains, after which the contribution of the matrix to fracture gradually increases. This is associated with the coalescence of cracks in neighbouring grains. At the end of loading (sample failure), the contribution of the grains, matrix, and the interface is ≈50%, ≈25%, and ≈25%, respectively. Hence, the contribution of grains to the total fracture of samples under dynamic loading is much greater than the contributions of the matrix and interfaces.

**Figure 16:**
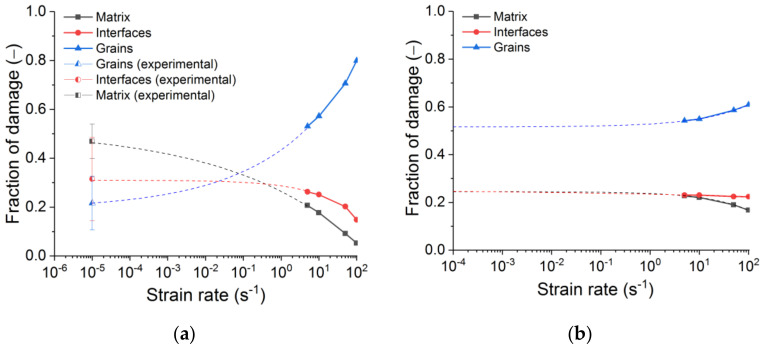
The dependences of the contributions of constituents to the fracture of refractory samples on the strain rate: tension (supplemented by experimental data) (**a**) and compression (**b**).

Figure 16 reveals how the contributions of constituents to the fracture of refractory samples alter with a strain rate increase. Here are the “final” values of fractions corresponding to fractured samples.

From experimental studies [12], the values of the quasi-static tensile crack path fractions per grains, matrix, and interfaces are notable for the considered refractory material. When comparing these data with the results of numerical simulation of dynamic tension (Figure 16a), one can witness significant changes in the fracture mechanisms during the transition from a quasi-static loading mode to a dynamic one. In the range of quasi-static strain rates (<10^−2^ s^−1^), the dominant contribution to fracture is made by damage in the matrix. Under dynamic loading, according to the results of numerical modelling, most of the damage is concentrated in large grains. The increasing contribution of the large grain damage with increasing strain rate is systematically seen for tension (Figure 16a) and compression (Figure 16b). Grain failure is observed throughout the sample. The effect is much more pronounced under tension. This intragranular damage is expected to be caused by secondary elastic waves from the forming main cracks.

To analyse the effect of the interface strength on the macroscopic dynamic strength of silica refractory, we carried out an additional numerical study with an interface strength that was 65% of the matrix strength (Table 4). The results confirmed that the difference between DIF for the models with the interface strength of 65% and 90% is less than 10% for compression and within 20% for tension—it is nearly constant at $\dot{\varepsilon }$ > 1 s^−1^. Thus, the obtained estimates of compressive DIF for refractory with interface strength 90% of the matrix strength (Figure 10b and Figure 12b) can be used as the “master curves”. Indeed, as the strength of the refractory interfaces changes, it is sufficient to measure the new UCS and UTS values of the samples under quasi-static loading. The dependences of the dynamic strength of the new material on the strain rate can be estimated with acceptable accuracy based on the use of the “master curves”.

## 4. Conclusions

The method of deformable discrete elements has been applied to model the meso-structural aspects of the failure of silica refractories. It has been discovered that the dynamic form of the combined Drucker–Prager–Rankine criterion is suitable to model the failure of quasi-brittle refractories under dynamic and quasi-static loading. This is explained by the importance of dynamic strengthening as well as for rather low rates of loading. A promising algorithm for determining the properties of microstructural constituents has been proposed. The algorithm is based on the scaling from the constituent of the best-known properties. The scaling factors are based on the Knoop hardness of the grains, matrix, and their interface. The validation and fine tuning of the properties were executed using the information on the fractions of the constituents in the real crack trajectories.

Compressive and tensile numerical tests were performed in the range of loading rates spanning the values typical for quasi-static laboratory tests, respective quasi-static in-service loads, and maximal dynamic in-service loads. From the numerical analysis, the following was concluded:

- An acceptable agreement was seen between the loading rate dependencies predicted by the model and average experimental trend typical for brittle and quasi-brittle materials. Particularly, the simulation results showed a non-linear increase of compressive and tensile strength of refractory with increasing strain rate. In both cases, the dynamic strength curves can be approximated by power functions with exponents significantly less than 1. The exponent for dynamic tensile strength is 1.4 times greater than the exponent for dynamic compressive strength. These results correlate well with experimental data on diverse heterogeneous ceramic-based materials, including concretes and rocks. Experimental data show that, first, the dependences DIF ($\dot{\varepsilon }$) are power functions with exponents usually around 0.5, and, second, the exponent for tensile DIF is up to two times greater than for compressive DIF [50,51,52,53]. The simulation results also indicate a transition from localised fracture (single macrocracks) to multiple fractures (branching network of mesocracks) and a progressive increase in the degree of sample fragmentation with increasing strain rate. The effect is especially pronounced at $\dot{\varepsilon }$ > 10^1^ s^−1^. This is also in good agreement with the dynamic test results of diverse quasi-brittle porous materials. Finally, it is pertinent to mention the increase in the number of broken mesoscopic hard inclusions at high strain rates observed in experiment and simulation (the trend from crack propagation around the inclusions to cutting them).
- The disproportional (power law) increase of the strength and fracture energy with the loading rates signals a reduced brittleness for the refractories under higher dynamic loading rates. On the microstructural level, this is seen by a more dispersed failure process and a higher fraction of large grain failure typically observed for higher rates of loading. A detailed experimental and computational study of this effect is the subject of further research.
- The compressive properties are more sensitive to the interface strength than the tensile properties. Under tension, a significant variation of the crack trajectory is observed despite the limited sensitivity of the global strength on the strength of the interface.
- The variation of the interface strength has a quantitatively similar effect on the quasi-static and dynamic compressive strength of the material.
- For tensile loading, the dependencies of strength on the loading rate for models with different interface strengths are less similar than those for compressive loading. This can indicate that the change in the contributions of different constituents to the sample damage with an increase in the tensile strain rate substantially depends on the static strength of the interfaces. A detailed analysis of this effect can be the subject of further research.

Refractory materials are designed to operate at high temperatures and temperature gradients. Therefore, thermal shock resistance is among the key service characteristics of refractories. The advantages of the method of uniformly deformable discrete elements make it possible to implement not only mechanical but coupled thermomechanical (for example, thermoelastic) models of quasi-brittle materials and take into account the thermal expansion of components and temperature dependence of component’s (local) strength. The development of such a model for silica refractory material and its application to study micro-scale mechanisms of formation of thermally induced cracks during thermal shocks is a priority topic for further research. The far-reaching goal of such research is the computer design of the multiscale internal structure of the refractory material to achieve the specified mechanical and thermophysical properties at high operating temperatures. The presented methodology of the development of a mesoscale mechanical model is a necessary initial stage of that research.

## Figures and Tables

**Table 1:** Initial values of mechanical characteristics of mesoscale components.

Properties	Grains	Matrix	Interfaces
Density (kg/m^3^)	2350	1670	Not applicable
Young’s modulus (GPa)	65	2.75	Not applicable
Poisson’s ratio (-)	0.2	0.2	Not applicable
$\mathrm{Tensile}\;\mathrm{strength}\;\sigma_{t}^{st}$ (MPa)	8.5	5.4	3.6
$\mathrm{Compressive}\;\mathrm{strength}\;\sigma_{c}^{st}$ (MPa)	34	21.6	14.4

**Table 2:** Defined parameters controlling the separation of elements.

Parameter	Grains	Matrix	Interfaces
*V_s_* (m/s)	1700	Not applicable	Not applicable
ε_max_	Not applicable	0.00025	0.00025
γ_max_	Not applicable	0.00025	0.00025

**Table 3:** Strength ratios.

Strength/Strength	Grains/Matrix	Grains/Interfaces	Matrix/Interfaces
σ*_t_*/σ*_t_*	1.574	2.361	1.5
σ*_c_*/σ*_c_*	1.574	2.361	1.5

**Table 4:** Final strength values.

Strength	Grains	Matrix	Interfaces
$\sigma_{c}^{st}$ (MPa)	680	432	288
$\sigma_{t}^{st}$ (MPa)	68	43.2	28.8

## Supplementary Materials

The following are available online at www.mdpi.com/article/10.3390/ma14237376/s1, Figure S1: The full-size images of Figure 6a–c.
