# Supplementary material for: Analysis of the Quasi-Static and Dynamic Fracture of the Silica Refractory Using the Mesoscale Discrete Element Modelling
Source: Materials (Basel). 2021 Dec 1;14(23):7376. doi: 10.3390/ma14237376 (PMC8658683; doi:10.3390/ma14237376)
Supplement: Supplementary file 1 [file materials-14-07376-s001.zip › materials-1418016-supplementary.pdf]

SUPPLEMENTARY MATERIALS

for

Analysis of the Quasi-Static and Dynamic Fracture of the Silica Refractory Using the Mesoscale Discrete Element Modelling

Aleksandr S. Grigoriev, Andrey V. Zabolotskiy, Evgeny V. Shilko, Andrey I. Dmitriev and Kirill Andreev

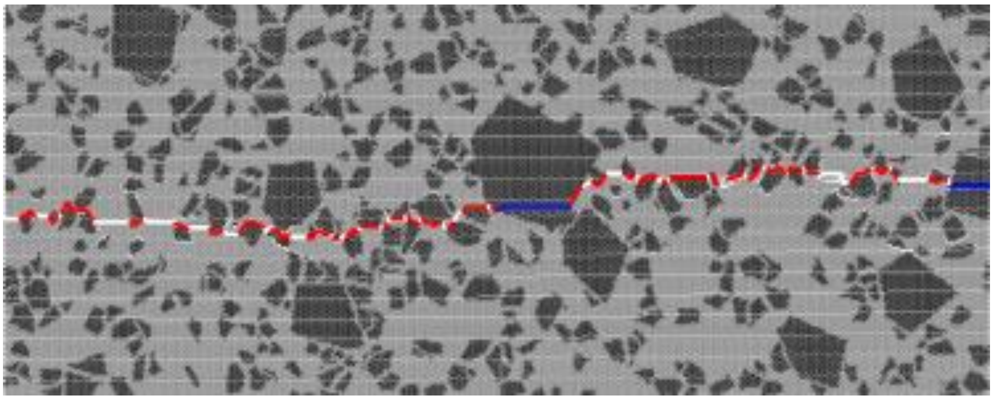

(a)

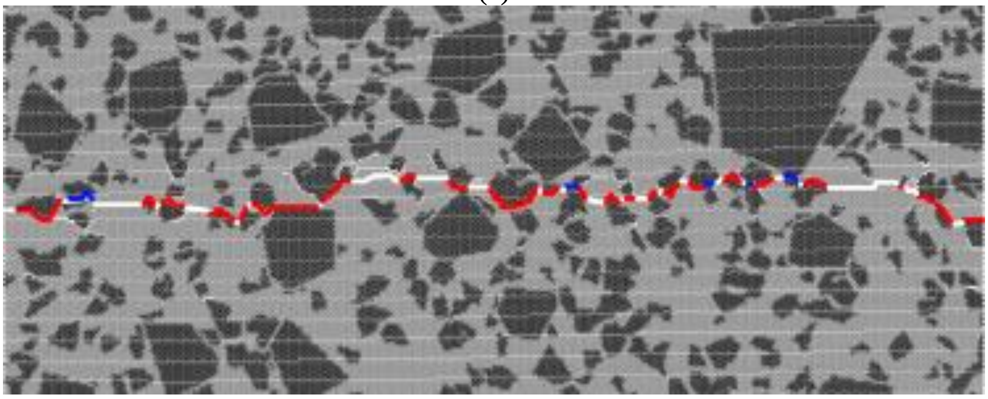

(b)

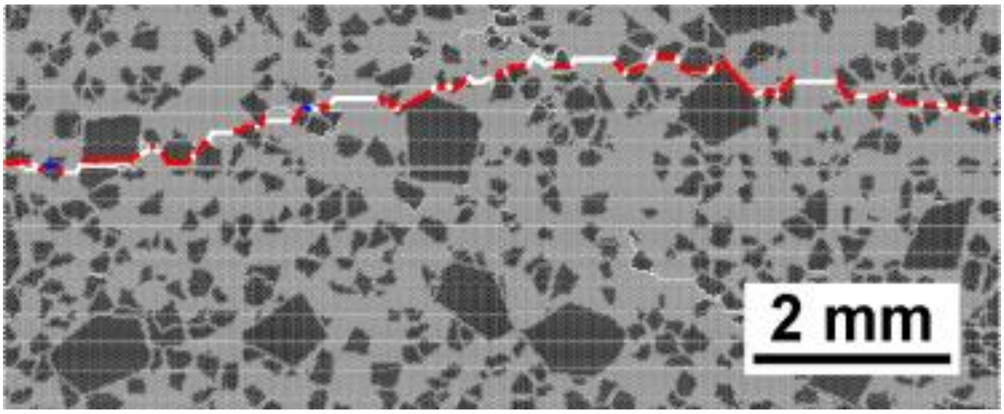

(c)

Figure S1. Crack patterns of three model samples under uniaxial tension. Quasi-static model of fracture. The interface's strength is 66.7% of the strength of the matrix. The above pictures are enlarged pictures in Figure 6a-c in the main body of the manuscript.
